# Recent Progress of SAPO-34 Zeolite Membranes for CO_2_ Separation: A Review

**DOI:** 10.3390/membranes12050507

**Published:** 2022-05-10

**Authors:** Muhammad Usman

**Affiliations:** Interdisciplinary Research Center for Hydrogen and Energy Storage (IRC-HES), King Fahd University of Petroleum & Minerals (KFUPM), Dhahran 31261, Saudi Arabia; muhammadu@kfupm.edu.sa

**Keywords:** zeolites, SAPO-34, membranes, CO_2_ separation, molecular sieving, CO_2_ mitigation

## Abstract

In the zeolite family, the silicoaluminophosphate (SAPO)-34 zeolite has a unique chemical structure, distinctive pore size, adsorption characteristics, as well as chemical and thermal stability, and recently, has attracted much research attention. Increasing global carbon dioxide (CO_2_) emissions pose a serious environmental threat to humans, animals, plants, and the entire environment. This mini-review summarizes the role of SAPO-34 zeolite membranes, including mixed matrix membranes (MMMs) and pure SAPO-34 membranes in CO_2_ separation. Specifically, this paper summarizes significant developments in SAPO-34 membranes for CO_2_ removal from air and natural gas. Consideration is given to a variety of successes in SAPO-34 membranes, and future ideas are described in detail to foresee how SAPO-34 could be employed to mitigate greenhouse gas emissions. We hope that this study will serve as a detailed guide to the use of SAPO-34 membranes in industrial CO_2_ separation.

## 1. Introduction

Anthropogenic activities are one of the primary causes of global warming. The primary cause of climate change is the combustion of fossil fuels, which results in enormous CO_2_ concentration in the atmosphere. Recently, the CO_2_ levels in the atmosphere were recorded as 414 parts per million, which is several folds higher than before the industrial revolution [1,2]. This increasing level of CO_2_ causes the greenhouse effect, contributes to respiratory disease, acts as asphyxiant, causes ocean acidification, and acts as a major source of energy imbalance due to a rise in earth temperature. Several approaches have been developed to mitigate CO_2_ in the atmosphere, including geological sequestration, catalytic conversion to useful products, adsorption, and membrane separation [3,4,5,6]. One approach is to separate and capture CO_2_ from air and its originating sources.

Several gas separation strategies have been independently researched for CO_2_ capture and separation, including cryogenic distillation and post-combustion processes such as absorption, adsorption, hydrated-based systems, and membrane separation techniques [7,8,9]. Cryogenic distillation necessitates huge distillation columns and is a high-energy process. Due to its compact footprint, simplicity, and great energy efficiency, membrane gas separation technology has been considered to be one of the most promising technologies to replace older technologies such as amine scrubbing. The membrane separation technique has considerable advantages over other separation technologies because it is a continuous separation process that consumes less energy, and the materials can be recycled. Recently, as compared with polymeric membranes, the incorporation of zeolites into polymers has been shown to improve CO_2_ separation performance significantly.

In 1756, Swedish scientist Axel Fredrik Cronstedt invented the term “zeolite” [10]. Zeolites have a unique chemical composition, distinctive pore size distribution, and chemical, thermal, and ion exchange properties [11,12,13,14,15,16,17,18,19,20,21]. These materials have been employed for a range of applications, including capture, purification, and catalysis [22,23,24,25,26,27,28,29,30]. Among zeolites, Lok et al. [31] introduced the family of SAPO zeolite materials. The SAPO-34 unit cell has chabazite (CHA)-type topology related to other aluminophosphates (such as SAPO-15, SAPO-11, SAPO-16, and SAPO-31), aluminosilicate (low-silica CHA and high-silica SSZ-13) and pure silicate (all-silica CHA). All these low and high-silica zeolite materials have been explored for gas separation applications [32,33,34,35,36].

**Figure 1:**
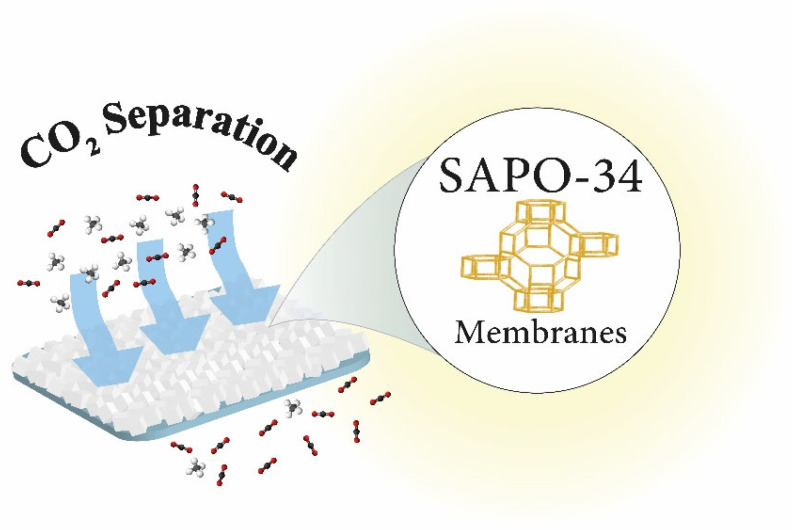
Schematic presentation of SAPO-34 membranes in CO_2_ separation.

**Figure 2:**
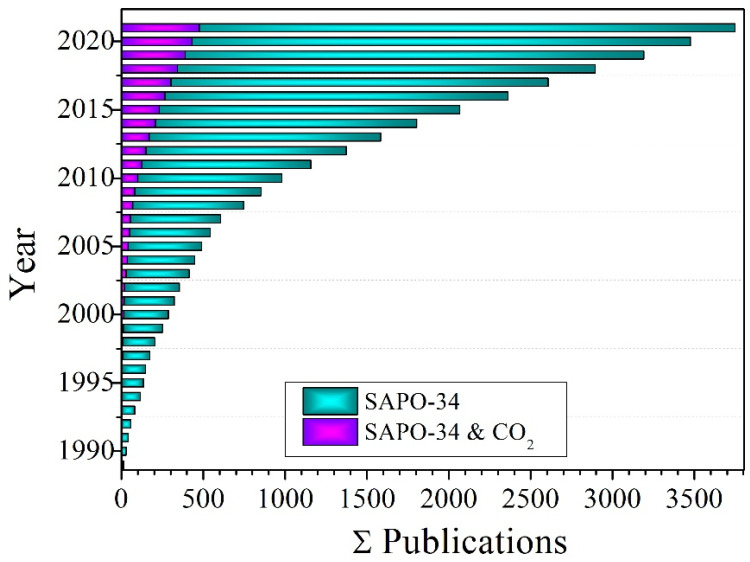
Histograms of SAPO-34 zeolite literature since 1990. Data were taken from SciFinder using keywords “SAPO-34” and “SAPO-34 and CO_2_”.

The SAPO-34 zeolite has intra-crystalline pore volumes and pore sizes ranging from 0.18 to 0.48 cm^3^/g and from 0.3 to 0.8 nm, respectively. The SAPO-34 structure is made up of eight-membered rings with a diameter of 9.4 (3.8 × 3.8), as shown in Figure 1. In addition to distinct pore size and volume, SAPO-34 material exhibits moderate to high hydrophobicity and has high thermal and hydrothermal stability. Due to these characteristic features, SAPO-34 has been extensively used in the methanol to olefin (MTO) process [37,38,39,40,41,42,43,44,45,46,47,48]. In recent years, SAPO-34 has been investigated for CO_2_ remediation, including CO_2_ capture [49,50,51], conversion [52,53,54], and separation [49]. Several studies have already covered a vast area of SAPO-34 materials research. Askari et al. [43] examined several synthetic procedures of SAPO-34. Ahmadi et al. summarized the deactivation of SAPO-34. Sun et al. looked at how to increase MTO performance in SAPO-34 by reducing the size of the zeolite using crystals and pore engineering. Furthermore, state-of-the-art SAPO-34 membranes and membranes reactor were addressed by Xu et al. [55]. Rimaz et al. proposed SAPO-34 as a best molecular sieve [56]. Kumar et al. studied CO_2_ capture by different zeolites in general, whereas Singh et al. [39] explored porous materials for CO_2_ capture applications. Kumar et al. [57] reviewed CO_2_ capture by various zeolites in general. Recently, Usman et al. [54] reviewed SAPO-34 zeolite materials for CO_2_ capture and conversion into useful chemicals. Despite the publication of multiple review studies, the role of SAPO-34 zeolite in CO_2_ mitigation has yet to be summarized. There has been a lot of research done on SAPO-34 membranes for CO_2_ separation, but no recent paper has been written about the role of SAPO-34 membranes in CO_2_ separation, especially from air (N_2_) and natural gas (CH_4_). Therefore, this review was conceived as a result of the rapidly developing research on SAPO-34, as shown in Figure 2, and its role in CO_2_ mitigation.

## 2. SAPO-34 Membranes for CO_2_ Separation

**Figure 3:**
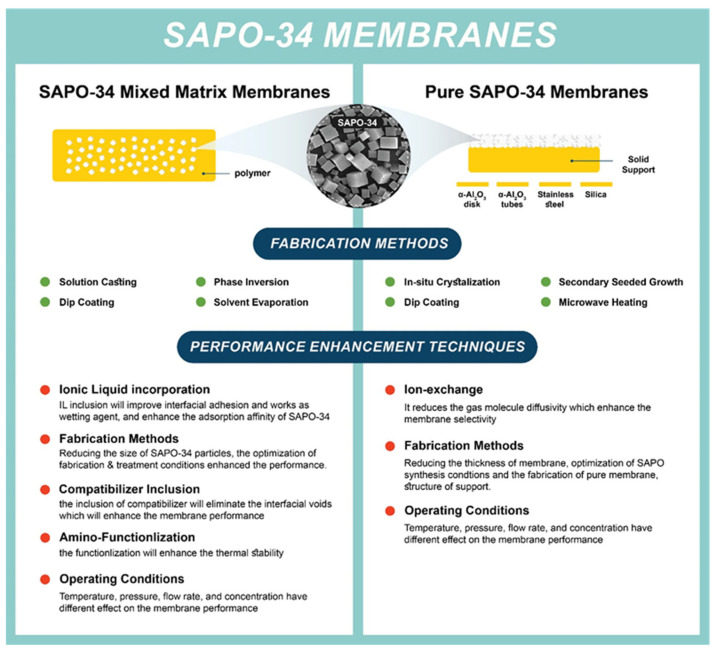
Schematic of fabrication method and performance advancement approaches for SAPO-34 membranes.

A crystalline hydrate aluminosilicate is distinguished by its uniform pore size (0.3–1.3 nm), as well as superior thermal, chemical, and mechanical stability. Zeolite is an ideal material for different applications, such as adsorption, ion exchange, and catalysis. Recently, it has become attractive for membrane applications due to its unique structure and excellent physicochemical properties [58]. Among more than 190 zeolite frameworks, a few have been distinguished for their promising separation performances. SAPO-34 is one of these structures that exhibits a significant separation performance, specifically removing CO_2_ from CH_4_ and N_2_ mixture. As shown in Figure 3, SAPO-34 membranes are mainly classified into two types: (1) Mixed matrix membranes (MMMs) where the SAPO-34 is incorporated in the polymer. These membranes are fabricated via various methods, including solution casting, phase inversion, solvent evaporation, and dip coating. (2) Pure SAPO-34 membranes where a substrate such as alumina, stainless steel, and silica are used as a support for the SAPO-34 membranes. Secondary seeded growth and in situ crystallization methods are the most frequent routes to fabricate these membrane types. In this review article, an extensive literature review about the separation and gas permeation of SAPO-34 membranes, in the last decade, is discussed including MMMs and pure membranes. 

### 2.1. SAPO-34-Based Mixed Matrix Membranes

Several gas separation technologies have been investigated for CO_2_ capture, including cryogenic distillation, post-combustion process, and membrane separation processes. Membrane separation technology shows significant merits as compared with other separation technologies because it is a continuous separation process, requires low energy consumption, and the materials can be regenerated. As compared with the polymeric membranes, MMMs that combine the advantages of both polymeric and inorganic materials have become the focus for a next-generation gas separation membrane. MMMs could provide a solution to the permeability and selectivity trade-off in polymeric membranes and bridge the gap with pure inorganic membranes. MMMs also offer the physicochemical stability of a ceramic material while ensuring the desired morphology with higher permeability, selectivity, hydrophilicity, fouling resistance, as well as greater thermal, mechanical, and chemical strength over a wider temperature and pH range. In MMMs where the flexibility, processability, and scalability of polymeric membranes meet the exceptional separation performance, the chemical and thermal stability of inorganic fillers have become a trending focus of academia and industry.

Since the first report of MMMs in 1970 [59], extensive research has been conducted to improve the separation performance and industrial implementation for a range of applications such as hydrogen recovery, treatment of natural gas, and air separation [60,61,62]. Different types of polymer matrices have been reported with various fillers, such as MOFs [63,64,65,66,67], COFs [68,69,70,71,72], carbon materials [73], zeolites [59], and other materials [74,75]. Zeolite materials with their sieving properties and cost-effectiveness on a large scale make them better candidates for gas separation. The most suitable zeolite filler for CO_2_ separation is SAPO-34 due to its unique structure and CO_2_ adsorption affinity [76].

#### 2.1.1. SAPO-34 MMMs

Peydayesh et al. [77] fabricated SAPO-34/Matrimid 5218 MMMs. They showed 55% and 97% enhancements in CO_2_ permeability and CO_2_/CH_4_ selectivity, respectively, indicating the good adhesion of the filler in the polymer matrix. Wu et al. [78] reported an MMMs obtained by the inclusion of SAPO-34 nanoparticles within a polyethersulfone (PESU) polymer. The separation performance increased with increasing SAPO-34 loading. In addition, the nanoparticle size was investigated, where 100 nm particles resulted in defective membranes. In contrast, 200 nm SAPO-34 showed fewer defects with a continuous interface and higher permselectivity than smaller particles. Carter et al. [79] reported three types of filler: SAPO-34, silica, and ZIF-8. Among all the fabricated MMMs, ZIF-8 showed the best performance owing to the strong interaction between the filler and polymer matrix and surface diffusion transport. In addition, the study claimed that pore size was the most influential factor in gas permeability, as it increased permeability. As a result of the reduced interfacial voids and chain mobility, the SPAO-34 MMMs showed high ideal selectivity. Messaoud et al. [80] reported a dip-coating route for fabricating SAPO-34/polyetherimide MMMs. This study investigated the effects of two solvents, N-methyl-2-pyrrolidone (NMP) and dichloroethane (DCE), on membrane fabrication. DCE resulted in better performance properties related to the entrapping of small DCE molecules in SAPO-34 particles, which induced the sealing of SAPO-34 pores. The best molecular sieving performance was achieved with 5 wt% SAPO-34 MMMs with 4.41 × 10^−10^ mol m^−2^ s^−1^ Pa^−1^ and 60 CO_2_/CH_4_ selectivity. Particle agglomeration was observed with 10 wt% MMMs. Zhao et al. [81] reported SAPO-34/Pebax1657 MMMs fabricated by solvent evaporation. The inclusion of SAPO significantly enhanced the CO_2_ permeability as compared with that of the neat membrane, whereas the selectivity remained constant. The effect of pressure was studied, and as a reason for plasticization, the permeation of MMMs increased with pressure. Junaidi et al. [82] conducted two studies on MMMs. First, asymmetric SAPO-34/PSf MMMs were prepared using the phase inversion method. The highest performance was achieved with 10 wt% MMMs with ideal selectivity 28.1 and 26.2 for CO_2_/CH_4_ and CO_2_/N_2_, respectively. When the filler loading was increased to >20 wt%, it led to poor interaction between filler and matrix which caused interfacial voids. They modified the SAPO-34 particles with the coupling agent APMS using two solvents, isopropanol and ethanol, before being added to the polymer matrix to overcome the previously reported challenge. The study showed that modified SAPO-34 MMMs exhibited better performance than unmodified and neat membranes owing to the reduction in interfacial voids [83]. An experimental and modeling study was reported by Santaniello et al. [76] where 200 nm SAPO-34 was incorporated, for the first time, in a polyhexafluoropropylene PHFP matrix. The MMMs with 24.6 v% and 36 v% showed an enhancement in the permeability and CH_4_/CO_2_ selectivity as compared with the neat membrane, which was ascribed to the increased polymer-free volume. The modeling part of gas transport confirmed the experimental results that 200 nm SAPO-34 particles provoked a polymer-free volume of 24.6 v%.

#### 2.1.2. SAPO-34 Functionalized MMMs

The functionalization strategy offers the prospect of enhancing membrane performance. Cakal et al. [84] reported the influence of compatibilizer additives on the permeation performance of SAPO-34/HMA/PES membranes. The elimination of interfacial voids in the membrane is the main role of the HMA compatibilizer. The improvement in CO_2_/CH_4_ selectivity for SAPO-34 (20 wt%)/HMA (10%)/PES as compared with neat PES was attributed to the reduction in the diffusion pathway of CH_4_. The effect of temperature was expected, as the permeability of all gases was enhanced as the temperature increased [85]. The effect of the functionalization of SAPO-34 with ethylenediamine (EDA) and hexylamine (HA) organic amino cations on the gas permeation, morphology, and pore size of SAPO-34/PES MMMs was investigated. The MMMs fabricated with modified SAPO-34 with the EDA agent showed better performance than the HA agent owing to higher amino grafting, which enhanced the filler/polymer adhesion, resulting in a better CO_2_/CH_4_ ideal selectivity [86]. Amino functionalization and ionic liquid inclusion were studied by Nasir et al. [87]. The study revealed that the improvement of the particles/polymer interphase was due to the incorporation of [emim][Tf_2_N] ionic liquids. Simultaneously, the amino functionalization of the SAPO-34 surface by EDA and HA enhanced the thermal stability of the MMMs. In addition, the membrane with the modified SAPO-34 and [emim][Tf_2_N] IL exhibited the best CO_2_/CH_4_ selectivity as compared with that of the neat membrane. The hydrophobicity of MMMs is a key factor for industrial applications. Functionalization of SAPO-34 using 1H,1H,2H,2H-perflourodecyltriethoxysilane (HFDS) fluorocarbons was reported by Junaidi et al. [88]. In this study, functionalized SAPO-34 particles were embedded in a PSf polymer to overcome the competitive adsorption of moisture under wet conditions. Among the fabricated MMMs, the SAPO-34 10 wt% + 0.1 HFDS/PSF membrane showed the best performance (CO_2_ permeance = 278 GPU) and (CO_2_/CH_4_ = 38.9) as compared with a bare polymer. The incorporation of modified SAPO-34 enhanced the membrane 17.64% hydrophobicity and showed better filler/polymer adhesion. In addition, SAPO-34 10 wt% + 0.1 HFDS/PSf membrane showed excellent stability for long-term stability tests under wet and dry conditions, whereas the unmodified membrane lost 90% of its performance under wet conditions.

Incorporating the third component in the MMM plays a vital role in improving SAPO-34 membrane performance. Nawar et al. reported the synergetic influence of ionic liquid (IL) inclusions on the separation of SAPO-34 MMMs [89]. In this study, 5 wt% SAPO-34 particles were incorporated into the polysulfone matrix, and the resulting membrane was immersed in 1-ethyl-3-methylimidazolium bis(tri-fluoromethylsulfonyl)imide IL. The membrane with the 0.2 M ionic liquid showed enhanced membrane performance as compared with the unmodified membrane, which was ascribed to interfacial defect reduction due to ionic liquid inclusion. Increasing the amount of ionic liquid caused a reduction in permeance and selectivity owing to pore and filler blockage. Ahmad et al. [90] used an [emim][TF2N] IL. Increasing the immersion time of SAPO-34 membranes led to an enhancement in the adsorption affinity of SAPO-34 for CO_2_ and filler/polymer interfacial adhesion, and SAPO-34 + IL/PSF showed the best performance as compared with neat PSF, with ideal selectivity of 20.35 and 18.82 for CO_2_/CH_4_ and CO_2_/N_2_, respectively. Mohshim et al. [91] reported the use of Tf2N in SAPO-34/PES. This work also proved that ionic liquids improve interfacial adhesion and function as wetting agents. The performance of the modified MMMs was significantly enhanced as compared with bare PES. A modeling study was conducted to study the effect of incorporating (emim [Tf_2_N]) and (emim[CF_3_SO_3_]) ionic liquids in a polymer matrix using the Maxwell, Lewis–Nielson, and Maxwell–Wagner–Sillar (MWS) gas separation models. The study showed a local agglomeration of SAPO-34 particles and a high deviation from the experimental results. Modification of the MWS model to include the wet phase factor showed good agreement with the experimental results [92]. Sen et al. [93] investigated the impregnation of carbon in polyetherimide using in situ carbonization to tailor SAPO-34 MMMs. Owing to the incompatibility, the impregnated carbon particles redecorated the interfacial pores formed between the filler and polymer. This approach minimized the interfacial pores/defects, which enhanced membrane performance.

#### 2.1.3. SAPO-34 MMMs and Operating Conditions

**Figure 4:**
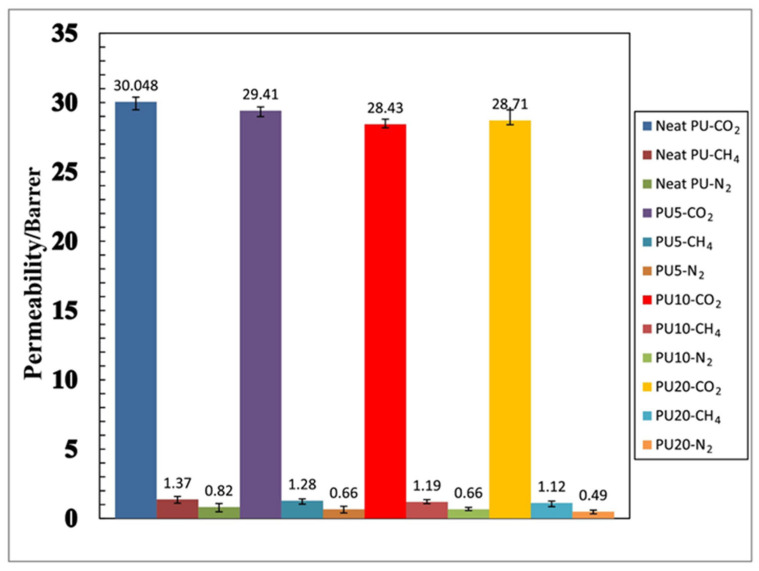
Effect of SAPO-34 content in the gas permeation properties of polyurethane–SAPO-34 membranes on the permeability of CO_2_, CH_4_, and N_2_ gases in 1.2 MPa pressure and selectivity of CO_2_/CH_4_ and CO_2_/N_2_ gases in 1.2 MPa pressure. Adapted with permission from Ref. [94]. Copyright 2019 Elsevier.

The operating conditions are one of the key factors affecting membrane performance. Sodeifian et al. [94] investigated the influence of pressure and inclusion of SAPO-34 nanoparticles. The study showed that increasing the pressure (0.4–1.4 MPa) caused an increase in CO_2_ permeability, whereas CH_4_ and N_2_ permeability remained constant. Increasing SAPO-34 within the polyurethane matrix decreases the permeability of CO_2_ and CH_4_ and enhanced the ideal selectivity of CO_2_/N_2_ and CO_2_/CH_4_, which indicated the benefit of SAPO-34 particle incorporation in the PU matrix, as shown in Figure 4.

Rabiee et al. [95] investigated the effects of temperature and pressure on the separation performance of SAPO-34/Pebax MMMs. The study showed that the incorporation of SAPO-34 led to an enhancement in gas permeability_,_ and the membranes exhibited diffusion-dominant behavior, and indicated the molecular sieving effect of SAPO-34. An increase in operating conditions and pressure (4–24 bar) led to an enhancement in gas permeation, increasing the driving force and solution diffusion mechanism. The temperature alternation also showed the same behavior, which increased the Pebax chain mobility around the filler. The above discussions are summarized in Table 1.

### 2.2. Pure SAPO-34 Membranes

The separation properties of mixed-matrix membranes for gas mixtures are unique because of their selective extraction of CO_2_, high efficiency, and flexibility. However, harsh operating conditions (high pressure and temperature) are applied to the membranes in real applications, leading to degradation of membrane performance. To overcome this challenge, the use of pure SAPO-34 as a zeolite membrane offers a promising route for achieving superior separation performance. In addition to its unique mechanical, thermal, and chemical stabilities, SAPO-34 is distinguished by competitive CO_2_ adsorption and extraordinary molecular sieving. However, the fabrication of pure SAPO-34 membranes requires a substrate to support the membrane. Stainless steel and alumina (disks, tubes, and hollow fibers) are the most frequently reported supports for pure SPAO membranes.

#### 2.2.1. SAPO-34/Alumina Membranes

**Figure 5:**
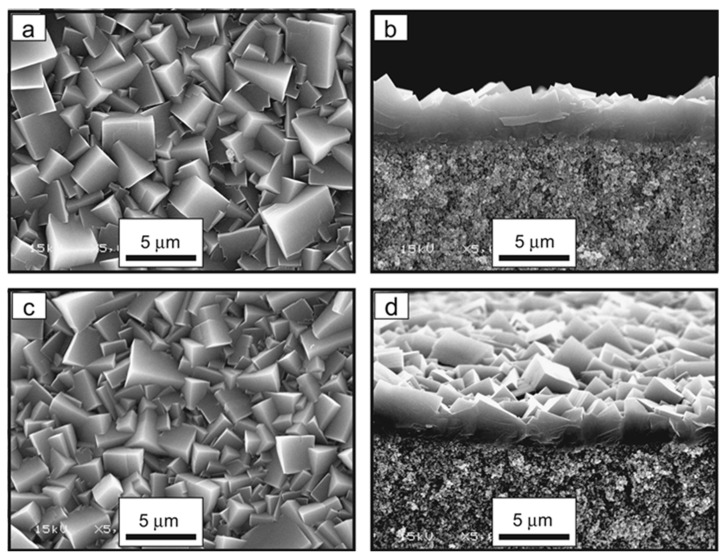
Surface and cross-sectional SEM images of SAPO-34 membranes prepared with Al(OH)_3_ and TEAOH/P_2_O_5_ ratios of 1.75 (**a**,**b**) and 2.0 (**c**,**d**). Adapted with permission from Ref. [99]. Copyright 2013 Elsevier.

Since the first paper on using alumina as a support for pure SAPO-34 membranes, extensive work has been conducted to improve membrane performance. The first SAPO-34/alumina membrane was reported in 1997 by Lixiong et al. [96]. A defect-free membrane was fabricated via in situ synthesis. The membrane exhibited a CO_2_ permeance of 29.9 × 10^−8^ mol/(m^2^ s Pa) with a CO_2_/N_2_ selectivity of 11.17. Poshusta et al. [97] reported SAPO-34 crystals supported on the inner walls of α-alumina tubes. The influences of temperature and pressure on single and mixed gases were investigated. The membrane showed CO_2_/CH_4_ ideal selectivities of 30 and 19 for mixed and single gases, respectively. Following a previous study, Poshusta et al. [98] fabricated another SAPO-34 membrane with a slight modification of the synthesis temperature. The membrane showed a high CO_2_ permeance for CO_2_ and a constant CO_2_/CH_4_ selectivity. Single gas permeation was governed by a molecular sieve, whereas mixed gas was governed by competitive adsorption. A facile, cost-effective, and less toxic waste synthesis method for SAPO-30/alumina membranes was reported by Zhou et al. [99]. The synthesis used only one template (TEAOH) and Al(OH)_3_ instead of Al(O-i-Pr)_3_. Figure 5 shows the microstructure analysis of SAPO-34 cubic crystals with a 1–3 μm crystal size and 3 μm membrane thickness. The formation of impermeable regions as a result of the synthesis gel was overcome by rinsing with deionized water for 24 h, followed by calcination. The membrane exhibited high separation properties for CO_2_. Carreon et al. reported an SAPO-34 membrane with a thinner SAPO layer (6–7.5 μm) fabricated using the secondary seeded growth method, which showed high performance in a single gas permeation [100].The performance enhancement was related to the small size of the zeolite crystals, structural properties of the support, and layer/support interactions. Li and Fan [101] investigated the modification of crystallization time. The reported membrane exhibited 1.2 × 10^−6^ mol/m^2^·s·Pa CO_2_ permeance with (CO_2_/N_2_ ratio of 32) a (50/50) mixture. A high selectivity stability was achieved under wet conditions with 8% water vapor and a 37% decline in CO_2_ permeance. Ion exchange was described as an effective route to enhance permeation performance by Chew et al. [102]. The microwave heating approach was reported to fabricate SAPO-34 membranes by ion-exchanging SAPO-34 with alkaline earth cations (Ba, Sr, Mg, and Ca). Ba-SAPO-34 showed the best result, with a 240% improvement in CO_2_/CH_4_ selectivity for equimolar mixture. The same group conducted a modeling study using a central composite design CCD and response surface methodology RSM to study the impact of the operating conditions on CO_2_ permeance and CO_2_/CH_4_ selectivity [103].

**Figure 6:**
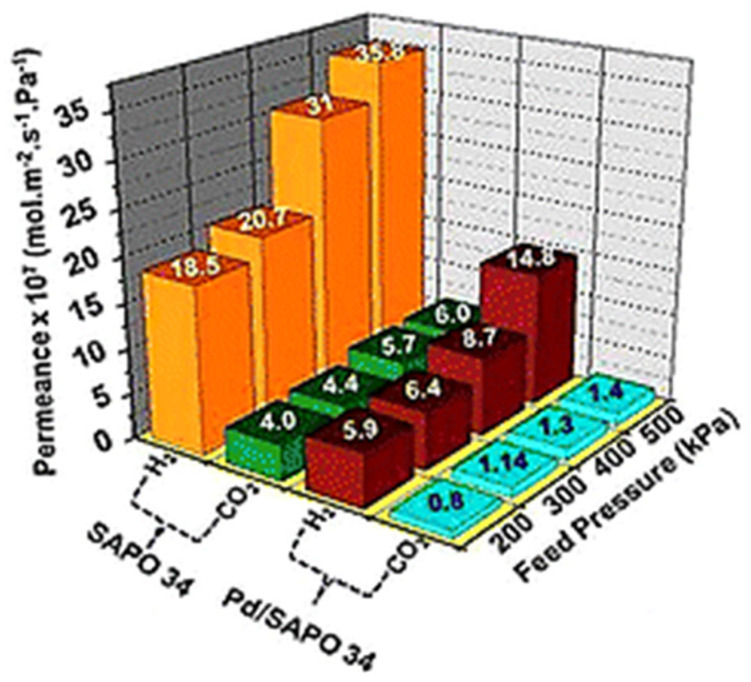
H_2_/CO_2_ separation performance on Pd/SAPO-34 membranes. Adapted with permission from Ref. [108]. Copyright 2019 Royal Society of Chemistry.

Furthermore, they investigated the separation performance at low CO_2_ concentrations. The membrane showed good stability and durability with CO_2_ permeance of 17.5 × 10^−7^ mol/m^2^·s·Pa and CO_2_/N_2_ selectivity of 78 for 5% CO_2_ on the feed side [104]. Li et al. [105] demonstrated the dip-coating effect of a thin layer of carbon/SAPO-34 using permeation analysis. This selectivity enhancement was ascribed to the permeation blockage of the non-absorbable gas. Shi et al. [106] reported, for the first time, a rapid free organic template synthesis method for SAPO-34 membrane on α-Al_2_O_3_ disk. The new approach enhanced the performance of membrane 1.2 times as compared with organic template membranes. The enhancement was attributed to the avoidance of defects in the membrane formed by the organic template after high-temperature calcination. SAPO-34/α-Al_2_O_3_ disk membranes were prepared by seeded growth using the microwave irradiation method for the first time by Liu et al. [107]. This synthesis route led to a homogenous, thin, and dense SAPO-34 layer on the α-alumina disk, which improved the separation properties. A strategy to prepare non-zeolitic pores on SAPO-34 membranes was highlighted by Das et al. [108] by the insertion of Pd nanoparticles. This strategy led to an enhancement in membrane quality for separation applications. As shown in Figure 6, the reduction in H_2_ permeance for Pd-SAPO-34 membranes as compared with unmodified membranes indicated the successful repair of non-zeolitic pores by Pd nanoparticles. However, the CO_2_ permeance remained almost stable at different feed pressures, confirming that the CO_2_ permeance on Pd-SAPO-34 membranes is a molecular sieving process. Thus, the selectivity for H_2_/CO_2_ was enhanced by up to 20.8 for Pd membranes as compared with unmodified membranes [108]. Wu et al. [109] studied the permeation analysis of (SSZ-13 and SAPO-34) membranes in a propane atmosphere. The orientation of SAPO-34 seed crystals played a more significant role in membrane performance than random orientation. 

Bing et al. [110] reported a spin-coated highly oriented SAPO-34 seed layer, and a well-intergrown oriented SAPO-34 membrane was fabricated by secondary microwave hydrothermal growth. This approach led to high-quality membranes with no large defects, which improved the membrane performance. Kgaphola et al. [111] reporteda nanocomposite SAPO-34 membrane fabricated by a pore-plugging hydrothermal PPH approach for post-combustion CO_2_ capture. The PPH approach provided high mechanical and thermal stability as compared with the hydrothermal and seeded growth methods. In addition, PPH minimized the high-temperature thermal expansion mismatch. Song et al. [112] fabricated a defect-free SAPO-34/alumina tube membrane by seeded growth under microwave irradiation. Different factors that affect the morphology and membrane structure were investigated, including the seed size, heating mode, aging, and synthesis time. The membrane performance under simulated power-plant flue gas was investigated by Liu et al. [113]. The membrane exhibited a high separation performance under dry conditions. However, 19.3% and 14.9% performance decline were observed under humid conditions. A facile defect healing strategy using vacuum-assisted deposition (VAD) for SAPO-34/alumina membranes was proposed by Mu et al. [114]. This strategy was effective in healing the nonselective defects of the membrane during preparation without affecting the CO_2_ permeance and significantly improving the CO_2_/CH_4_ selectivity. A bis(triethoxysilyl)ethane (BTESE)-derived organosilica was used for healing. In addition, the effects of pressure, temperature, and healing cycles were investigated. More cycles of healing via VAD caused a decrease in the membrane performance, indicating the low CO_2_ permeance and selectivity of BTESE. Recently, Wang et al. [115] fabricated highly oriented SAPO-34 nanofilms with ordered channels (prepared by the gel-nuclei-less method) on α-Al_2_O_3_ tubes via a secondary growth route. The superior performance was related to the large and continuous area of the nanofilms, which provided a fast permeating and CO_2_ selective route.

#### 2.2.2. SAPO-34/Stainless Steel Membranes

Li et al. reported the in situ crystallization of SAPO-34 membranes using tubular stainless steel with 0.8 μm pores. A CO_2_/CH_4_ selectivity of 270 and a CO_2_ permeance of 2.4 × 10^−7^ mol/(m^2^ s Pa) were achieved [116]. They also studied the influence of impurities (N_2_, H_2_O, C_2_H_4_, C_3_H_8_, and n-C_4_H_10_) on the membrane performance [117]. This study revealed that N_2_ had an insignificant impact on membrane performance. The introduction of light HCs caused a decline in permeation performance, which was related to the higher heat of adsorption in C_4_. However, reversible performance recovery was observed after removing impurities. The ratio of (Si/Al gel) was investigated by Li et al. [118] where a membrane with a Si/Al gel ratio of 0.15 showed the highest CO_2_ permeance and selectivity for CO_2_/CH_4_. Venna et al. [119] fabricated oriented thin SAPO-34/stainless-steel membranes via a seeded growth approach. This method was the main reason for the high membrane performance CO_2_/CH_4_ selectivity of 9 and CO_2_ permeance of 2.5 × 10^−6^ mol/(m^2^ s Pa). Functionalization of SAPO seeds with different organic amino cations (EDA, HA, and octylamine) has been reported. The EDA-functionalized membrane showed high performance as compared with the unfunctionalized membrane and other modified membranes. The preferential adsorption of CO_2_ was a key factor for performance improvement.

#### 2.2.3. SAPO-34/Silica Membranes

Silica can also be used as a substrate in SAPO-34 membranes. Makertihartha et al. [120] reported a defect-free SAPO-34 membrane fabricated via two methods: optimized secondary growth (combined rubbing/aging) and rubbing. The first approach showed higher CO_2_/N_2_ selectivity, which was attributed to the defect-free and continuous membrane layer. Owing to the concentration polarization phenomenon at higher pressures, the membrane exhibited two N_2_ flux regions. Table 2 summarizes the application of pure SAPO-34 membranes in natural gas purification and air separation.

#### 2.2.4. Scale-Up and Industrial Approach of Pure SAPO-34 Membranes

In practical applications, it is necessary to develop membranes with high performance and low fabrication costs. Hollow fiber supports have been demonstrated to be a better alternative to disk and tubular supports. A few attempts have shown promising results in the scalability aspect of SAPO-34 membranes, including the study that conducted by S. Li and coworkers [101]. The study demonstrated the scalability of SAPO-34 membrane on a porous tubular stainless-steel support from 5 cm to 25 cm, which was optimized. Crystallization time, gel composition, and precursor were optimized to acquire the same membrane performance of 5 cm SAPO-34 membrane. Additionally, the fabrication cost was reduced by using low-cost aluminum sources. Despite the challenges in scalability of pure SAPO-34 membranes, hollow fiber-based route offers an alternative approach for scalability [126]. Recently, B. Wang et al. [50] reported a simple and controllable scale-up fabrication method for SAPO-34 on commercial α-alumina tubular support. The dilution of mother liquid had a vital impact in the synthesis method. The membrane exhibited ultra-high CO_2_ permeance and CO_2_/CH_4_ selectivity as compared with the typical membrane.

Chen et al. [123] fabricated an SAPO-34 membrane on α-Al_2_O_3_ four-channel hollow fibers (4CHF) via a secondary growth method. An excellent performance was achieved, which was related to the transfer resistance of the support. Furthermore, the synthesis parameters (temperature and time) were investigated. The study showed that the synthesis temperature improved the separation selectivity at the expense of the CO_2_ permeance, which was attributed to the increasing thickness. Furthermore, the synthesis time showed that membrane performance increased with time. However, at a certain point, the reverse effect was observed, which might have been related to the crystal dissolution of the zeolite layer. Rehman et al. reported a facile strategy to overcome the degradation of SAPO-34 membrane performance by water interaction [124]. It was based on the dip coating of the membrane in α-, ω-dihydroxypolydimethylsiloxane (PDMS) to provide surface protection. In addition to protection, the modification enhanced the membrane performance under wet and dry conditions as compared with unmodified membranes. In addition, the study showed that PDMS protected the membrane, and the performance of the membrane did not change too much (5–8%) after half-year storage, although a 71% decline in CO_2_ permeance and 85% in CO_2_/CH_4_ selectivity were observed for unmodified membrane. Another report by Rehman et al. investigated a hydrophobic modification of the SAPO-34/4 CHF membrane. The study reported the use of n-dodecyltrimethoxysilane as a surface modifier for membranes with superior selectivity as compared with unmodified membranes under wet conditions [125]. Ping et al. reported a scale-up study [121] where SAPO-34 was prepared by seeded-gel synthesis on seven-channel monolithic alumina supports. They systematically studied the effects of different monolithic supports (single and seven channels) and different seeding methods (dip coating, rub coating, and seeded gel). The seeded gel on the seven-channel monolith support showed the best performance, which was ascribed to the permeate flow rates through the monoliths. In addition, the seed-gel approach reduced the cost and time required to prepare zeolite membranes.

## 3. Summary and Outcome

In the last decade, extensive research progress in SAPO-34 membranes has led to high-performance membranes. The development of molecular sieve membranes made of SAPO-34 zeolite for CO_2_ separation from the air and natural gas has made great progress. Pure inorganic and MMMs are summarized in this review. Based on the literature, SAPO-34 membranes are mostly studied for possible applications in natural gas purification and its separation from air. Previously, it has also been studied for hydrogen separation and catalytic membrane reactors for elevated temperature reactions. However, a number of directions have to be investigated and studied to further improve the SAPO-34 membranes for industrial applications. Thus, there is still room for boosting the performance of SAPO-34 membranes. This review highlights the following research viewpoints for further studies:

1. Rigid pore membranes and ion-exchange membranes must be developed to increase the solubility and rejection of particular gases.
2. More studies should be focus on the transport mechanism of MMMs and pure SAPO-34 membranes.
3. Regarding techno-economic analysis, the economics of each separation method must be evaluated in terms of factors such as cost per kilogram of product and energy consumption per kilogram, with a view to encourage and explore the research in this direction.
4. In hydrogen separation using Pd-based membrane, SAPO-34 interlayer can play an important role as a diffusion barrier and substrate modifier over the support. SAPO-34 membranes were prepared on α-Al_2_O_3_ four-channel hollow fiber (4CHF) supported by secondary growth method. Moreover, the 4CHF supported SAPO-34 membranes could also provide high membrane packing density for membrane modules and cut fabrication costs, which is a promising candidate for practical applications. Therefore, more work is required to explore its application in Pd-based membranes by using vacuum-assisted seeding and secondary growth methods on the different substrates.
5. Regarding He separation applications, SAPO-34 membranes are less explored in helium separation from He/CH_4_ since this is one the most attractive separations; due to size sieving and diffusivity difference, SAPO-34 membrane surpassed the Robeson limit upper bound, making these membranes appealing for the recovery of helium from natural gas.
6. In natural gas purification, SAPO-34 is one of the best candidates due to its unique pore size and adsorption capabilities. However, it decomposes over the time (years) due to the presence of moisture in the raw natural gas. Therefore, it is recommended to improve its moisture resistance property by modifying its surface using hydrophobic barrier. The chemical modification strategy of SAPO-34 would strengthen the properties of the membranes. More studies of this type are required in SAPO-34 membrane research for boosting membrane steadiness and performances under high humidity conditions.
7. Regarding defect-free membranes, thin SAPO-34 membranes over the supports have been produced through various synthetic strategies to reduce defects; however, it is extremely challenging to obtain defect-free membranes. Many studies have been attempted to heal these defects using post-treatments, but some methods are costly and some result in producing thicker membranes which ultimately lower the permeance. Therefore, more work is required to heal the defects of SAPO-34 membranes by carefully designing the post-synthetic modifications that should balance the resultant membranes’ cost and permeance.
8. The demand of the scaling up of mixed matrix membranes is a hot topic. However, the commercialization of the SAPO-34-based MMMs is still in the early stage and requires more work to develop facile synthetic methods with cost effective techniques. Scaling up the Pure SAPO-34 membranes is a pivotal requirement for industrial commercialization. However, systematic optimization of the fabrication parameter is required to acquire the scaling-up procedure. Pure SAPO-34 thin membranes are produced on solid supports. However, they face many challenges when it comes to scaling-up their fabrication on longer tubular supports (more than 20 cm). In large-scale production, these membranes might require better approaches such as modified synthetic schemes and big autoclaves, etc. These approaches are reviewed in the above sections. The scalability of SAPO-34 membranes and mixed matrix membranes are still in the early stage and need more investigation and development in the scalability aspect before reaching the commercialization stage. Thus, more efforts are needed to produce longer SAPO-34 membranes before being implemented at an industrial scale. Hollow-fiber-based module systems could provide an alternative pathway owing to their large area to volume ratios.
9. The high cost of the SAPO-34 zeolite membrane modules as compared with the polymeric membranes is one barrier to implementing these membranes in the industry for real practical applications. The cost in synthesizing SAPO-34 or zeolite membranes comes mainly from templates, Al/Si sources, and supports. A limited number of research studies have reported an alternative synthesis approach using low-priced alumina and/or template-free synthesis. The entire membrane cost consists of the zeolite filler and membrane support. As a result, research activities should consider choosing and fabricating less expensive membrane supports. Therefore, more studies are required to seek a cost-effective synthesis approach for SAPO-34 membranes to achieve cost-effective, scalability, and stability SAPO-34 membranes. In addition, improvement of SAPO-34 membrane toward industrial implementation at high operating conditions should be considered.

## Figures and Tables

**Table 1:** Summary of separation performance for SAOP-34 MMMs.

Filler	Substrate	CO_2_ Permeance	CO_2_/CH_4_ Selectivity	CO_2_/N_2_ Selectivity	Ref.
Neat	Matrimid 5218	4.4 Barrer	34	-	[77]
SAPO-34 2 wt%	Matrimid 5218	4.5 Barrer	41.98	-	[77]
SAPO-34 5 wt%	Matrimid 5218	4.6 Barrer	44.24	-	[77]
SAPO-34 10 wt%	Matrimid 5218	5.3 Barrer	50.82	-	[77]
SAPO-34 15 wt%	Matrimid 5218	5.9 Barrer	58.14	-	[77]
SAPO-34 20 wt%	Matrimid 5218	6.9 Barrer	66.99	-	[77]
Neat	Polyethersulfone (PESU)	6.7 Barrer	37.8	-	[78]
SAPO-34 NP 20 wt%	Polyethersulfone (PESU)	8.2 Barrer	42.6	-	[78]
SAPO-34 NP 30 wt%	Polyethersulfone (PESU)	8.9 Barrer	48.3	-	[78]
Neat	Matrimid 5218	9.5 ± 1.07 GPU	29.81	13.63	[79]
SAPO-34 10 wt% uncalcined	Matrimid 5218	7.63 ± 0.81 GPU	31.79	26.31	[79]
SAPO-34 10 wt% calcined	Matrimid 5218	12.5 ± 1.3 GPU	9.32	10.50	[79]
Neat	Polyetherimide	6 × 10^−10^ mol/(m^2^ s Pa)	0.02	-	[80]
SAPO-34 5 wt%	Polyetherimide	4.4 × 10^−10^ mol/(m^2^ s Pa)	60	-	[80]
SAPO-34 10 wt%	Polyetherimide	6 × 10^−10^ mol/(m^2^ s Pa)	8	-	[80]
Neat	Pebax 1657	100 Barrer	16.7	53.8	[81]
SAPO-34 23 wt%	Pebax 1657	134 Barrer	21.7	55.2	[81]
SAPO-34 33 wt%	Pebax 1657	252 Barrer	17	55	[81]
SAPO-34 50 wt%	Pebax 1657	339 Barrer	16.8	53.2	[81]
Neat	Polysulfone (Asymmetric)	22.0 ± 3.42 GPU	17.3	16.5	[82]
SAPO-34 5 wt%	Polysulfone (Asymmetric)	205.9 ± 7.26 GPU	22.5	21.4	[82]
SAPO-34 10 wt%	Polysulfone (Asymmetric)	314.0 ± 4.65 GPU	28.2	26.1	[82]
SAPO-34 20 wt%	Polysulfone (Asymmetric)	281.18 ± 6.92 GPU	10.9	10.7	[82]
SAPO-34 30 wt%	Polysulfone (Asymmetric)	232. ± 3.21 GPU	3	2.9	[82]
Neat	Polysulfone (Asymmetric)	105 GPU	15	13	[83]
SAPO-34 10 wt%	Polysulfone (Asymmetric)	459 GPU	27	21	[83]
SAPO-34E 10 wt%	Polysulfone (Asymmetric)	706 GPU	31	28	[83]
SAPO-34I 10 wt%	Polysulfone (Asymmetric)	775 GPU	28	22	[83]
Neat	Polyhexafluoropropylene (PHFP)	290 Barrer	14.1	-	[76]
SAPO-34 NP 24.6 v%	Polyhexafluoropropylene (PHFP)	468 Barrer	15.8	-	[76]
SAPO-34 NP 36 v%	Polyhexafluoropropylene (PHFP)	437 Barrer	17.5	-	[76]
Neat	PES	4.45 Barrer	33.2	-	[84]
HMA 10%	PES	0.8 Barrer	32.3	-	[84]
SAPO-34 20 wt%	PES	5.7 Barrer	37	-	[84]
SAPO-34 20 wt% + HMA 10%	PES	1.3 Barrer	44.7	-	[84]
HMA 4%	PES	5.1 Barrer	39.3	-	[85]
SAPO-34 20 wt%	PES	13.8 Barrer	32.7	-	[85]
SAPO-34 20 wt% + HMA 4%	PES	7.8 Barrer	41.6	-	[85]
SAPO-34	PES	18 GPU	1.2	-	[86]
SAPO-34 20 wt%	PES	30 GPU	1.3	-	[86]
SAPO-34 20 wt% m-EDA	PES	10.0 GPU	12.14	-	[86]
SAPO-34 20 wt%	PES	50 GPU	2.5	-	[87]
SAPO-34 20 wt%/IL	PES	0.03 GPU	4.9	-	[87]
SAPO-34 20 wt% m-EDA/IL	PES	0.09 GPU	26.5	-	[87]
SAPO-34 20 wt% m-HA/IL	PES	0.045 GPU	37.2	-	[87]
Neat	Polysulfone (PSf)	21.3 ± 2.8 GPU	17.2	-	[88]
SAPO-34 10 wt%	Polysulfone (PSf)	317.0 ± 3.5 GPU	27.9	-	[88]
SAPO-34 20 wt%	Polysulfone (PSf)	283.0 ± 2.2 GPU	10.8	-	[88]
SAPO-34 10 wt% + 0.5 wt%HFDS	Polysulfone (PSf)	310.4 ± 1.7 GPU	30.4	-	[88]
SAPO-34 10 wt% + 1 wt%HFDS	Polysulfone (PSf)	278.8 ± 2.1 GPU	38.9	-	[88]
SAPO-34 10 wt% + 1.5 wt%HFDS	Polysulfone (PSf)	259.7 ± 4.2 GPU	37.3	-	[88]
SAPO-34 20 wt% + 0.5 wt%HFDS	Polysulfone (PSf)	332.1 ± 5.5 GPU	11.9	-	[88]
SAPO-34 20 wt% + 1 wt%HFDS	Polysulfone (PSf)	293.7 ± 4.9 GPU	27.5	-	[88]
SAPO-34 20 wt% + 1.5 wt%HFDS	Polysulfone (PSf)	306.8 ± 5.2 GPU	24.8	-	[88]
SAPO-34 5 wt%	Polysulfone (PSf)	6.1 GPU	4.9	5.1	[90]
SAPO-34 5 wt%/IL(0.2 M)	Polysulfone (PSf)	24.89 GPU	35.06	40.15	[90]
Neat	Polysulfone (PSf)	5.60 ± 0.75 GPU	3.24	6.15	[91]
SAPO-34 5 wt%	Polysulfone (PSf)	6.53 ± 1.22 GPU	3.47	5.67	[91]
SAPO-34 5 wt%/IL(0.4 M)	Polysulfone (PSf)	4.82 ± 1.28 GPU	4.86	8.04	[91]
SAPO-34 5 wt%/IL(0.6 M)	Polysulfone (PSf)	7.24 ± 1.78 GPU	20.35	18.82	[91]
SAPO-34 20 wt%	Polyethersulfone (PES)	85.7 GPU	20.67	-	[92]
SAPO-34 20 wt% + IL 5 wt%	Polysulfone (PSf)	230.8 GPU	-	46.20	[92]
SAPO-34 20 wt% + IL 10 wt%	Polysulfone (PSf)	255.69 GPU	-	58.83	[92]
SAPO-34 20 wt% + IL 15 wt%	Polysulfone (PSf)	279.2 GPU	-	60.62	[92]
SAPO-34 20 wt% + IL 20 wt%	Polysulfone (PSf)	300.0 GPU	-	62.58	[92]
Neat	Polyetherimide	3.8 × 10^−10^ mol/(m^2^ s Pa)	-	2.23	[94]
SAPO-34 10 wt%	Polyetherimide	2.8 × 10^−8^ mol/(m^2^ s Pa)	-	2.54	[94]
SAPO-34 25 wt% + Carbonization	Polyetherimide	8.42 × 10^−8^ mol/(m^2^ s Pa)	-	6.47	[94]
SAPO-34 40 wt%	Polyetherimide	9.1 × 10^−7^ mol/(m^2^ s Pa)	-	5.05	[94]
Neat	Polyurethane	30.05 Barrer	21.93	36.64	[89]
SAPO-34 NP 5 wt%	Polyurethane	29.41 Barrer	22.97	44.56	[89]
SAPO-34 NP 10 wt%	Polyurethane	28.43 Barrer	23.89	54.67	[89]
SAPO-34 NP 20 wt%	Polyurethane	28.71 Barrer	25.63	58.59	[89]
Neat	Pebax 1074	120 Barrer	17.5	60.3	[95]
SAPO-34 5 wt%	Pebax 1074	123 Barrer	18.5	61	[95]
SAPO-34 10 wt%	Pebax 1074	130 Barrer	22	62.5	[95]
SAPO-34 20 wt%	Pebax 1074	152 Barrer	29	68	[95]
SAPO-34 30 wt%	Pebax 1074	156 Barrer	35	69	[95]

**Table 2:** Summary of separation performances for pure SAOP-34 membranes.

Filler	Substrate	CO_2_ Permeance	CO_2_/CH_4_ Selectivity	CO_2_/N_2_ Selectivity	Ref.
SAPO-34	Stainless steel	2 × 10^−7^ mol/(m^2^ s Pa)	270	-	[116]
SAPO-34 (M1)	Stainless steel	1.1 × 10^−7^ mol/(m^2^ s Pa)	27	-	[117]
SAPO-34 (M2)	Stainless steel	1.4 × 10^−7^ mol/(m^2^ s Pa)	54	-	[117]
SAPO-34 (M3)	Stainless steel	1.4 × 10^−7^ mol/(m^2^ s Pa)	87	-	[117]
SAPO-34 (M3)	Stainless steel	4.9 × 10^−8^ mol/(m^2^ s Pa)	55	-	[117]
SAPO-34	Stainless steel tube	1.2 × 10^−7^ mol/(m^2^ s Pa)	170	-	[118]
SAPO-34	Stainless steel	2.52 × 10^−6^ mol/(m^2^ s Pa)	9.30	-	[119]
SAPO-34 (nonfunctionalized)	Stainless steel	4.6 × 10^−7^ mol/(m^2^ s Pa)	159	29	[119]
SAPO-34 (0.15 mmol of HA)	Stainless steel	3.7 × 10^−7^ mol/(m^2^ s Pa)	238	36	[119]
SAPO-34 (0.15 mmol of OA)	Stainless steel	1.9 × 10^−7^ mol/(m^2^ s Pa)	229	30	[119]
SAPO-34 (0.15 mmol of ED)	Stainless steel	5 × 10^−7^ mol/(m^2^ s Pa)	245	39	[119]
SAPO-34	α-Al_2_O_3_ disk	6.40 × 10^−8^ mol/(m^2^ s Pa)	-	4.16	[96]
SAPO-34	α-Al_2_O_3_ disk	29.9 × 10^−8^ mol/(m^2^ s Pa)	-	11.17	[119]
SAPO-34	α-Al_2_O_3_ tubes	2.4 × 10^−8^ mol/(m^2^ s Pa)	19	5.7	[97]
SAPO-34	α-Al_2_O_3_ tubes	15.5 × 10^−8^ mol/(m^2^ s Pa)	20	7.1	[98]
SAPO-34	α-Al_2_O_3_	1.2×10^−6^ mol/(m^2^ s Pa)	70	-	
SAPO-34	α-Al_2_O_3_	1.8 × 10^−6^ mol/(m^2^ s Pa)	171	-	[100]
SAPO-34	α-Al_2_O_3_	1.2 × 10^−6^ mol/(m^2^ s Pa)	-	32	[101]
SAPO-34	α-Al_2_O_3_	0.45 × 10^−6^ mol/(m^2^ s Pa)	-	9.5	[101]
SAPO-34	α-Al_2_O_3_	0.7 × 10^−7^ mol/(m^2^ s Pa)	-	10	[101]
Ba-SAPO-34	α-Al_2_O_3_ tubes	37.6 × 10^−8^ mol/(m^2^ s Pa)	103	-	[102]
Ba-SAPO-34	α-Al_2_O_3_ tubes	17 × 10^−8^ mol/(m^2^ s Pa)	36	-	[102]
SAPO-34	α-Al_2_O_3_ disk	17.5 × 10^−7^ mol/(m^2^ s Pa)	-	78	[104]
SAPO-34	α-Al_2_O_3_ disk	8.1 × 10^−7^ mol/(m^2^ s Pa)	-	25.1	[104]
Thin carbon/SAPO-34	α-Al_2_O_3_ tubes	8.7 × 10^−8^ mol/(m^2^ s Pa)	87	-	[105]
SAPO-34	seven-channel monolith Al_2_O_3_	3.7 × 10^−7^ mol/(m^2^ s Pa)	21	-	[121]
SAPO-34	seven-channel monolith Al_2_O_3_	6.3 × 10^−7^ mol/(m^2^ s Pa)	44	-	[121]
SAPO-34	seven-channel monolith Al_2_O_3_	6.3 × 10^−7^ mol/(m^2^ s Pa)	56	-	[121]
SAPO-34	α-Al_2_O_3_ disk	1.63 × 10^−6^ mol/(m^2^ s Pa)	258	-	[106]
SAPO-34	α-Al_2_O_3_ disk	1.72 × 10^−6^ mol/(m^2^ s Pa)	213	-	[106]
SAPO-34	α-Al_2_O_3_ disk	1.26 × 10^−6^	95	-	[107]
SAPO-34	α-Al_2_O_3_ tubes	1.5 × 10^−6^ mol/(m^2^ s Pa)	100	-	[109]
SAPO-34	α-Al_2_O_3_ tubes	5 × 10^−7^ mol/(m^2^ s Pa)	100	-	[109]
SAPO-34	α-Al_2_O_3_	1.57 × 10^−6^ mol/(m^2^ s Pa)	109	-	[110]
SAPO-34	α-Al_2_O_3_ tubes	2.44 × 10^−7^ mol/(m^2^ s Pa)	-	7.9	[111]
SAPO-34	α-Al_2_O_3_	6.2 × 10^−7^ mol/(m^2^ s Pa)	89	-	[112]
SAPO-34	α-Al_2_O_3_ tubes	1.82 × 10^−6^ mol/(m^2^ s Pa)	-	32.9	[113]
SAPO-34	α-Al_2_O_3_ tubes	4.67 × 10^−7^ mol/(m^2^ s Pa)	-	10.3	[113]
SAPO-34	α-Al_2_O_3_ tubes (untreated)	2.5 × 10^−7^ mol/(m^2^ s Pa)	61	-	[114]
SAPO-34	α-Al_2_O_3_ tubes (treated)	2.4 × 10^−7^ mol/(m^2^ s Pa)	158	-	[114]
SAPO-34	α-Al_2_O_3_ tubes	27 ± 1.41 × 10^−7^ mol/(m^2^ s Pa)	146 ± 5.6	-	[122]
SAPO-34	α-Al_2_O_3_ tubes	1.2 × 10^−5^ mol/(m^2^ s Pa)	135	41	[115]
SAPO-34	α-Al_2_O_3_/(4CHF)	1.18 × 10^−6^ mol/(m^2^ s Pa)	160	-	[123]
SAPO-34	α-Al_2_O_3_/(4CHF)	2.3 × 10^−7^ mol/(m^2^ s Pa)	53		[124]
SAPO-34/PDMS	α-Al_2_O_3_/(4CHF)	1.18 × 10^−7^ mol/(m^2^ s Pa)	68	-	[124]
SAPO-34/PDMS	α-Al_2_O_3_/(4CHF)	4.2 × 10^−7^ mol/(m^2^ s Pa)	86	-	[124]
SAPO-34	α-Al_2_O_3_/(4CHF)	1.7 × 10^−8^ mol/(m^2^ s Pa)	0.9	-	[125]
SAPO-34/PDMS	α-Al_2_O_3_/(4CHF)	1.18 × 10^−6^ mol/(m^2^ s Pa)	160	-	[125]
SAPO-34	Silica tubes	2.01 × 10^−6^ mol/(m^2^ s Pa)	-	53	[120]
SAPO-34	Silica tubes	2.01 × 10^−6^ mol/(m^2^ s Pa)	-	2.08	[120]

## Data Availability

Not applicable.
